# Effectiveness of percutaneous electrolysis in subacute and early chronic medial gastrocnemius muscle injuries: a single-blinded randomized clinical trial

**DOI:** 10.3389/fmed.2026.1757222

**Published:** 2026-03-13

**Authors:** Carlos Vicente-Vega, Fausto José Barbero-Iglesias, Javier Martín-Vallejo, Sergio Varela-Rodríguez

**Affiliations:** 1 Programa de Doctorado en Salud, Discapacidad, Dependencia y Bienestar, Universidad de Salamanca, Salamanca, Spain; 2 Department of Nursing and Physiotherapy, University of Salamanca, Salamanca, Spain; 3 Department of Statistics, University of Salamanca, Salamanca, Spain; 4 Institute of Biomedical Research of Salamanca (IBSAL), Salamanca, Spain; 5 Fisiostudio Clinic, Salamanca, Spain

**Keywords:** fibrosis, gastrocnemius injury, invasive physiotherapy, muscle scar tissue, percutaneous electrolysis, rehabilitation, tennis leg

## Abstract

**Background:**

Chronic injuries of the medial gastrocnemius muscle are clinically relevant conditions that often result in persistent pain, functional limitations, and delayed return to activity. Although conservative management is usually prescribed, outcomes are not always satisfactory, and minimally invasive approaches such as percutaneous electrolysis (PE) have been proposed as potential therapeutic alternatives.

**Methods:**

This randomized clinical trial included 71 patients with medial gastrocnemius injuries of at least 3 weeks' duration. Participants were randomly allocated to high-intensity PE (*n* = 23), low-intensity PE (*n* = 23), or a sham control group (*n* = 25). Participants received three sessions, once per week for three consecutive weeks. Outcomes were pain during gait and contraction, fatigue, kinesiophobia, and ankle range of motion (ROM), assessed at baseline and after the intervention.

**Results:**

High-intensity PE produced significant reductions in pain during gait and contraction compared with the sham group (*p* < 0.05). Low-intensity PE was associated with a significant reduction in fatigue compared with sham (*p* = 0.024). No significant between-group differences were observed in kinesiophobia or range of motion. Within-group analyses showed improvements in several outcomes among patients treated with PE (all, *p* < 0.05).

**Conclusions:**

Percutaneous electrolysis may effectively reduce pain during gait and contraction, as well as fatigue, compared with a sham intervention in patients with subacute to early chronic medial gastrocnemius injuries.

**Clinical trial registration:**

https://clinicaltrials.gov/study/NCT06713200, identifier: NCT06713200.

## 1 Introduction

Following injury, the development of fibrous scar tissue is commonly associated with an overproduction of extracellular matrix components, excessive proliferation of fibroblasts and myofibroblasts, and increased collagen synthesis (1, 2). Muscle healing typically progresses through inflammatory, proliferative, and remodeling phases. In the initial stages, scar tissue exhibits an immature composition (3). Subsequently, type III collagen is progressively deposited, reaching its maximum concentration before being gradually replaced by type I collagen, accompanied by structural reorganization that enhances the tissue's ability to withstand mechanical loads (2). However, when fibrotic remodeling becomes excessive or dysregulated, the resulting scar tissue may become stiffer and less elastic than healthy muscle tissue (4). Mechanically, this increased rigidity generates abnormal tensions in surrounding tissues, elevating the risk of microtears or recurrent injuries in the affected area (5). From a neuromuscular perspective, inadequate healing may impair proprioception and motor control, reducing the muscle's capacity to coordinate contractions and appropriately absorb mechanical loads during physical activity (6). These neuromuscular impairments, combined with unfavorable biomechanical changes, predispose the athlete to reinjury, often resulting in longer recovery periods compared with primary injuries (7, 8).

This scenario presents a challenge in clinical practice, as there is currently no standard treatment protocol for addressing this type of muscle injury. Management strategies often depend on factors such as the location and severity of the injury, as well as the individual characteristics of the patient. Rehabilitation typically focuses on restoring movement and ensuring appropriate load management (9, 10). However, in cases where recovery is unsatisfactory, adjuvant therapies have been introduced particularly during the subacute and chronic phases, such as shockwave therapy (11, 12), platelet-rich plasma (13, 14), low-level laser (15), growth factor and cell therapies (16), and percutaneous electrolysis (PE) (17). Despite their growing use, the clinical evidence supporting many of these interventions remains heterogeneous, with variable methodological quality and inconsistent findings, particularly in the context of muscular fibrotic lesions. This lack of consensus highlights the need for well-designed clinical trials to clarify the role of emerging interventions such as PE.

PE is a minimally invasive physiotherapy technique that applies galvanic current through a needle to induce a controlled inflammatory response, thereby promoting the regeneration of damaged tissues (17). This therapeutic approach has promising potential in facilitating scar tissue remodeling. At the cathodic needle application site, PE induces a transient pro-inflammatory response during the first days, characterized by increased expression of cytokines, as well as the recruitment of macrophages and M1 polymorphonuclear cells (18–20). This initial phase is followed by a regulatory anti-inflammatory response, together with stimulation of neoangiogenesis and activation of matrix remodeling mechanisms, including matrix metalloproteinases, VEGF, and PPAR-γ, which collectively contribute to the degradation of excess extracellular matrix and the synthesis of type I collagen (19–21). In addition, PE has been shown to enhance the expression of genes involved in tissue repair and regeneration, creating a favorable environment for muscle healing (21). Taken together, these biological responses provide a mechanistic rationale for the clinical application of PE in fibrotic musculoskeletal conditions (22, 23). Although most previous studies have focused on tendinous tissue, growing evidence suggests that similar regenerative mechanisms may operate in skeletal muscle.

Experimental studies in animal models have shown that PE can modulate the inflammatory response and promote regeneration. Abat et al. (24) induced gastrocnemius injury in rats and observed reductions in pro-inflammatory cytokines and increases in anti-inflammatory mediators and vascular endothelial growth factor, supporting enhanced angiogenesis and tissue repair. Similar findings were reported by Santafé and Margalef and Valera-Garrido et al. (25, 26), who demonstrated accelerated muscle recovery and improved neuromuscular activation after galvanic current or PE treatment. Moreover, Jordá et al. (27) confirmed decreased IL-6 and chemokines and increased IL-10 and IL-13 levels, reinforcing the hypothesis that PE favors skeletal muscle regeneration through local immune modulation. Clinical reports have also yielded promising results. Abat et al. (28) described successful recovery after PE treatment in a pectoralis major injury, while Valera-Garrido et al. and Jiménez-Rubio et al. (29, 30) reported early return-to-play in elite athletes with rectus femoris lesions. De-la-Cruz-Torres et al. (31) investigated chronic soleus injuries in female football players and found greater pre-post improvements in pain and functional parameters in the PE group compared with exercise alone, although between-group differences did not reach statistical significance. Collectively, these preclinical and clinical findings suggest that PE may promote muscular repair by modulating inflammatory and regenerative processes. Given the shared fibrotic characteristics between tendinous and muscular scar tissue, the present study aimed to evaluate the clinical effectiveness of percutaneous electrolysis (PE) in subacute to early chronic medial gastrocnemius lesions. We hypothesized that both high- and low-intensity PE (3 and 0.3 mA, respectively) would be more effective than a sham intervention in reducing pain and improving function in adults with subacute to early chronic medial gastrocnemius injuries. Accordingly, the primary objective was to determine the short-term effectiveness of PE at two different intensities compared with a sham procedure. The outcome variables analyzed included pain (during gait and muscle contraction), range of motion (ROM), muscle fatigue, and kinesiophobia.

## 2 Materials and methods

### 2.1 Study design

A prospective, single-blinded, randomized controlled trial with three parallel groups (high-intensity PE, low-intensity PE, and sham PE) was conducted between November 2024 and March 2025 to evaluate the effects of percutaneous electrolysis (PE) on fibrotic scar tissue in patients with subacute and early chronic medial gastrocnemius injuries. The intervention consisted of three sessions, delivered once per week over three consecutive weeks. Both baseline and post-intervention assessments were performed.

This report follows the Consolidated Standards of Reporting Trials (CONSORT) 2010 statement (32). The study was conducted in accordance with the ethical principles of the Declaration of Helsinki. The protocol was reviewed and approved by the Research Ethics Committee of the Salamanca Health Area (ID: PI 2023 08 1418-TD) and was prospectively registered at ClinicalTrials.gov (ID: NCT06713200).

All participants provided written informed consent before enrollment. The document described the study objectives, required data, participants' rights, and the procedures involved.

### 2.2 Participants, recruitment, and eligibility criteria

Between November 2024 and March 2025, participants were recruited through social media and email outreach to different Andalusian sports federations to ensure broad dissemination among individuals potentially meeting the eligibility criteria. In addition, patients referred through other healthcare channels were also screened. All candidates underwent an initial interview and ultrasound assessment to confirm compliance with the selection requirements. All participants were recreationally active adults engaged in habitual physical activity, with no inclusion of elite or professional athletes. NSAID and analgesic use was discouraged and verbally monitored at each session.

The inclusion criteria considered individuals aged between 18 and 65 years presenting a medial gastrocnemius muscle injury with a duration of at least 3 weeks. A lesion was classified as subacute to early chronic when its duration was ≥3 weeks. This time frame corresponds to the early remodeling phase of muscle healing, during which fibrotic processes begin to develop (approximately 21–28 days post-injury) (33). The injury was considered eligible if one of the following was present: (a) a medical diagnosis of medial gastrocnemius fiber rupture confirmed by ultrasound, or (b) ultrasound evidence of scar tissue consistent with muscle healing. Ultrasound diagnosis was based on altered echotexture with focal hyperechoic areas and preserved muscle fiber continuity, without evidence of acute disruption. Ultrasound examinations were performed by a physiotherapist with 5 years of experience in musculoskeletal sonography. Additional criteria included the presence of pain in the affected area, provision of written informed consent, and availability for follow-up throughout the study period. Exclusion criteria included ongoing rehabilitation for other musculoskeletal disorders, belonephobia (fear of needles), fibromyalgia, and uncontrolled metabolic diseases.

### 2.3 Sample size calculation

Given the absence of previous randomized clinical trials evaluating this intervention in chronic medial gastrocnemius injuries, the sample size calculation was based on studies conducted in other musculoskeletal conditions using PE. An effect size of *f* = 0.25 (moderate magnitude) was adopted according to Cohen's conventions for medium effects (34) and supported by previous randomized controlled trials investigating PE in musculoskeletal disorders (31, 35, 36), which reported moderate between-group differences in pain and functional outcomes, with power levels around 80% and confidence levels of 95%. A statistical power of 95% (1–β = 0.95) and a significance level of α = 0.05 were established *a priori* to minimize the risk of type II error, ensuring a high probability of detecting a true effect given the expected moderate-to-low magnitude. The sample size was computed using G^*^Power 3.1 (Heinrich-Heine-Universität Düsseldorf, Düsseldorf, Germany) software for a two-way mixed ANOVA (between-subjects factor: three groups; within-subjects factor: two measurements—pre- and post-intervention), assuming a correlation among repeated measures of 0.50 and a sphericity correction factor (ε = 1).

The calculation indicated a required sample size of 66 participants. To ensure adequate statistical power and to compensate for potential attrition, which is frequently observed in invasive and potentially painful interventions, the recruitment target was increased to 75 participants as a preventive strategy. No interim analyses were conducted, and the decision to increase recruitment was made independently of outcome data. This approach was adopted to preserve methodological rigor and statistical validity.

### 2.4 Allocation and randomization

The sample was randomized at a 1:1:1 ratio using EPIDAT version 4.2 (Dirección Xeral de Saúde Pública – Xunta de Galicia and PAHO/WHO, Santiago de Compostela, Spain) software, using a randomized block design. As patients were enrolled, they were assigned to an intervention group according to the generated sequence, ensuring property random allocation into one of the three study groups: (A) high-intensity PE; (B) low-intensity PE; (C) sham PE.

The randomization sequence was generated by an independent researcher not involved in the study procedures, who also prepared individually numbered, opaque, and sealed envelopes containing the group assignment. Each envelope was opened only after the baseline evaluation of the participant, thereby maintaining allocation concealment throughout the recruitment process.

### 2.5 Blinding

A single-blind design was implemented so that neither participants nor the outcome assessors were aware of group assignment. However, the clinician responsible for administering the interventions could not be blinded, as the clinician was required to apply PE at different intensities or perform the sham procedure. This clinician did not participate in any other aspect of the study, such as data collection or analysis, which were carried out by an independent investigator.

In the sham group, needle insertion was performed following the same protocol as in the active groups. The device was activated to reproduce the same operational sounds and procedure, but the intensity and duration parameters were set to zero, ensuring that no galvanic current was delivered. Impedance levels were visually monitored on the display to confirm the absence of electrical output. Consequently, participants did not experience the typical galvanic sensation; however, as they had been informed beforehand that the perception of current could vary between individuals, the effectiveness of blinding was preserved. Blinding effectiveness was not formally assessed, but no participant reported awareness of their group assignment.

### 2.6 Interventions

This randomized clinical trial included three treatment groups, each receiving three once-weekly sessions over three consecutive weeks. Before the application of PE or sham procedure, all participants received a standardized pre-treatment protocol. This protocol consisted of: (1) 10 min of manual massage therapy applied to the areas surrounding the lesion within the triceps surae region, focusing on tissues adjacent to the medial gastrocnemius injury, using longitudinal and transverse strokes with mild-to-moderate pressure; (2) 5 min of subtalar joint mobilization performed in supine position using graded oscillatory techniques within pain-free range; and (3) 10 min of radiofrequency diathermy [Diacare 7000 (distributed by Fisaude S.L., Fuenlabrada, Madrid, Spain), CE 0476] applied in capacitive mode at 0.5 MHz over the medial gastrocnemius region, with intensity adjusted to achieve a tolerable thermal sensation without discomfort. This protocol was predefined and applied uniformly across all groups to replicate routine clinical practice conditions and to ensure baseline procedural homogeneity before the experimental intervention.

**Figure 1:**
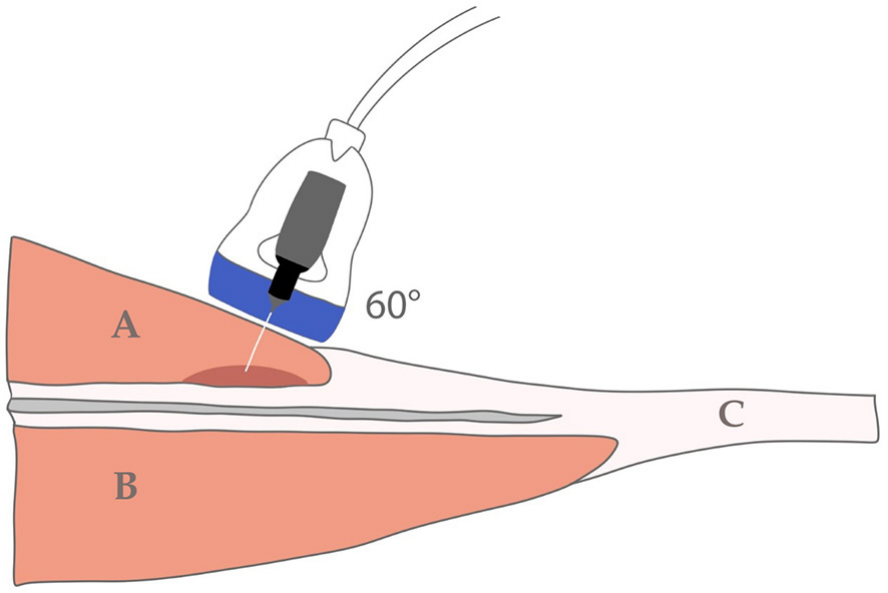
Angle of needle insertion in the medial gastrocnemius. **(A)** Medial gastrocnemius. **(B)** Soleus. **(C)** Achilles tendon.

During PE intervention, patients were placed in the prone position with ankle support for comfort. The technique was performed using a CE-marked medical device (EPI Alpha, 0051, Barcelona, Spain) and a sterile, disposable stainless steel needle (0.25 mm diameter). Needle length was selected based on the patient's anatomical characteristics (generally 40 or 50 mm). Following the protocol described by Bagcier and Yurdakul (37) and the aseptic recommendations, the needle was inserted at a 60° angle to the skin, as illustrated in Figure 1.

In the high-intensity PE group, the needle remained inserted for 90 s, with a 3 mA galvanic current applied during the final 24 s. This current was delivered in 3-s pulses, each separated by a 5-s pause, and the sequence was repeated three times, as described in previous studies on PE (36, 38, 39).

The low-intensity PE group followed the same procedure in terms of material, positioning and session frequency. However, the galvanic current was set to 0.3 mA and applied continuously for 90 s, as described in previous studies on PE (36, 38).

In the sham PE group, the needle remained inserted for 90 s and the device was activated to emit the characteristic operating sound, but no current was administered.

Across all groups, the needle remained inserted for the same duration (90 s). The only variable that differed between groups was the dosage of galvanic current applied, allowing for an isolated evaluation of its effects. Adverse events were monitored during and after each treatment session. Participants were systematically asked about increased pain, discomfort, or any unexpected reactions.

### 2.7 Outcomes

Two assessments were conducted: the first immediately before the intervention and the second 28 days after the first session, corresponding to approximately 1 week after completing the 3-week intervention period. No long-term follow-up was performed. The evaluator responsible for data collection was blinded to participant's group allocation. For the final assessment, participants were contacted solely for outcome assessment. In addition to the primary and secondary outcomes presented below, sociodemographic data, including age, body mass index and sex were also collected.

#### 2.7.1 Primary outcome

The primary outcome variable was pain perception during gait, assessed using an 11-point Numeric Pain Rating Scale (NPRS) where 0 indicates no pain and 10 indicates the maximum tolerable pain. Participants were asked to walk at their usual pace (approximately 4–5 steps) and then report the level of discomfort experienced during ambulation. This scale has demonstrated high test–retest reliability in people with chronic pain (ICC = 0.96) (40).

#### 2.7.2 Secondary outcomes

The secondary outcomes included pain during contraction, kinesiophobia, ankle dorsiflexion and triceps surae fatigue.

1. Pain during contraction was evaluated using an 11-point NPRS where 0 indicates no pain and 10 indicates the maximum tolerable pain. Subjects were asked to rate their discomfort while maintaining a bilateral heel-raise position. A score of 10 was assigned if participants were unable to hold the position for at least one complete elevation. This scale has shown excellent test-retest reliability in people with chronic pain (ICC = 0.96) (40).
2. Level of kinesiophobia was measured with the Spanish-validated Tampa Scale of Kinesiophobia short version (TSK-11SV), which assesses fear of movement or re-injury. It consists of 11 items, with scores ranging from 11 to 44. This questionnaire has demonstrated excellent test-retest reliability in chronic pain populations (ICC = 0.91) (41).
3. Ankle dorsiflexion range of motion (ROM) was assessed through the angle of dorsiflexion with the knee extended. This test primarily reflects the flexibility of the triceps surae, particularly the gastrocnemius muscle, rather than pure joint or capsular mobility (42). The test was performed barefoot, with the tested foot aligned to a marked midline on the floor and the contralateral foot placed one step forward. The endpoint was defined as the extent of 7/10 discomfort on a verbal numerical pain rating scale (NPRS), without heel lift or pelvic rotation. This criterion has been shown to yield high intra-session reliability when assessing triceps surae flexibility (42). Participants maintained knee and hip extension and used the wall for support (43). A digital camera [Canon EOS 2000D 24.1 MP (Canon Inc., Tokyo, Japan)], reflective markers, and motion analysis software (RULER, Polytechnic University of Valencia) were used to record the ankle dorsiflexion angle (44). This method has demonstrated high test-retest reliability for assessing triceps surae flexibility (ICC = 0.92) (42).
4. Triceps surae muscle fatigue was evaluated with a unilateral heel-raise test. Participants stood on one leg, with a slight external hip rotation and trunk aligned horizontally (45), and performed rhythmic heel raises following a metronome set at 40 beats per minute (Garmin Fenix 5/5S). Fatigue was defined as failure to achieve full heel elevation for three consecutive beats (46). Light fingertip support on a wall was permitted to maintain balance. This test has shown excellent test–retest reliability in participants with chronic pain (ICC = 0.96) (47).

### 2.8 Statistical analysis

Qualitative variables were described using frequencies and percentages, while quantitative variables were described using means and standard deviations. Normality was assessed using the Shapiro-Wilk test for both pre-intervention values and pre- and post-intervention differences. Pre-post differences were calculated to analyze whether the mean change differed between intervention groups. To compare the change, a one-way ANOVA was performed when the assumptions of homoscedasticity and normality were met; otherwise, the non-parametric Kruskal–Wallis test was used. The *post hoc* tests used were the *t*-test in the case of ANOVA and Dunn's test in the case of the Kruskal–Wallis test. The Bonferroni correction was performed in both *post hoc* tests. The unbiased estimator of the standardized mean difference and its standard error were calculated. Confidence intervals were calculated at 95%, and the significance level was set at 5%. Data analysis was performed using IBM-SPSS 22.0 package (Armonk, NY). Statistical analyses were conducted using a per-protocol (complete-case) approach, as missing outcome data were minimal (< 6%) and balanced across groups.

## 3 Results

### 3.1 Baseline characteristics of the participants

**Figure 2:**
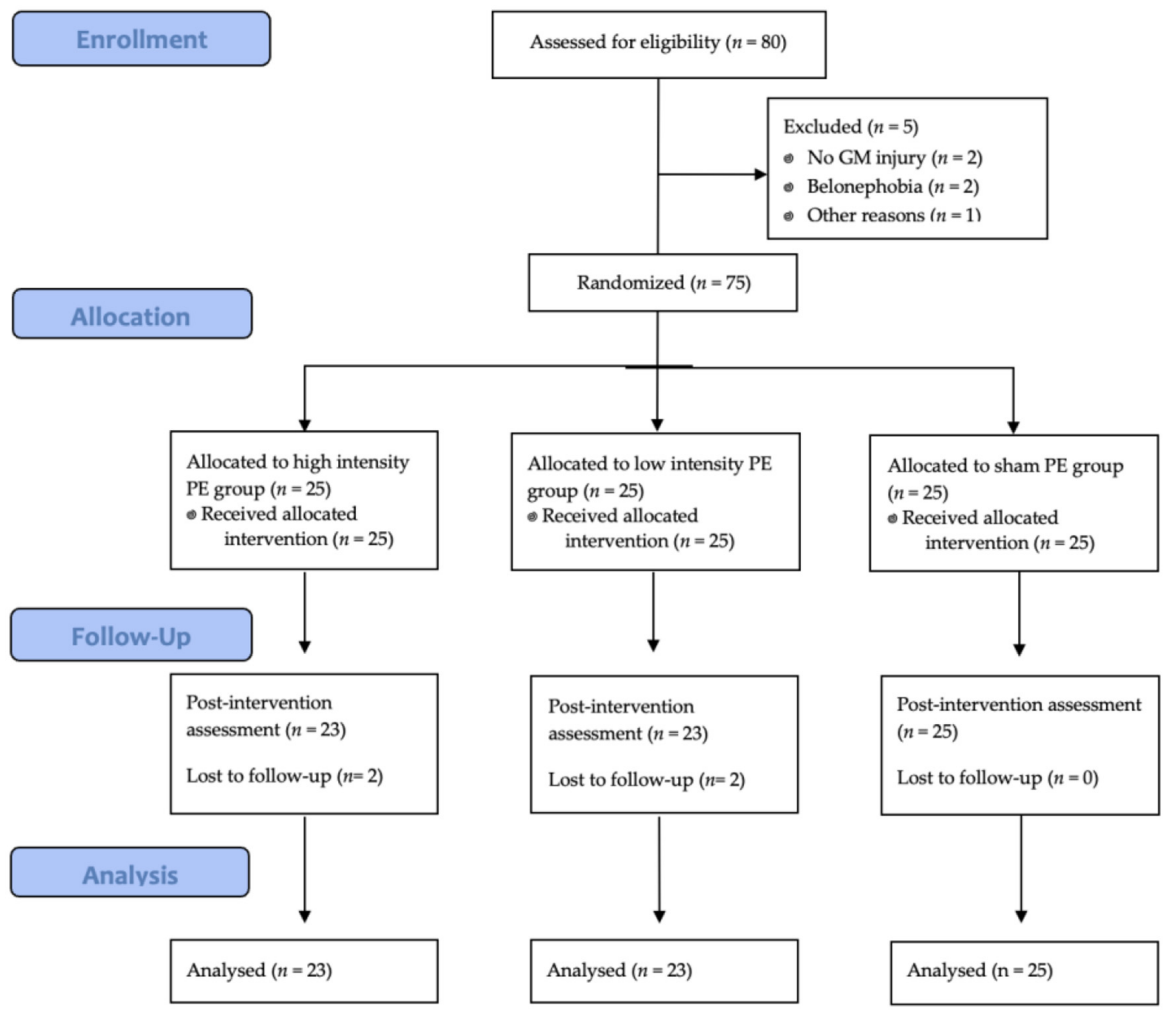
CONSORT flow diagram of participants.

Eighty individuals were screened for eligibility, of whom seven were excluded because they did not meet the eligibility criteria. Finally, 75 participants were enrolled, and 71 completed the protocol (32 women and 39 men). A per-protocol analysis was conducted, including only participants who completed all intervention sessions and both pre- and post-treatment assessments. Participants were randomly allocated into three groups: two with 23 participants and one (sham PE) with 25. Figure 2 shows the participant flow chart, including the two dropouts. All remaining participants completed all three sessions, as well as the pre- and post-treatment assessments, with no adverse effects recorded.

A descriptive analysis of sociodemographic variables was conducted to characterize the sample and assess potential baseline differences between groups. Table 1 presents the baseline characteristics of the participants.

**Table 1:** Baseline demographic and outcome measures.

**Variable**	**Total**	**High-intensity PE**	**Low-intensity PE**	**Sham PE**	***p-*value**
**Demographic**					
Age (years)	45.26 ± 10.78	47.65 ± 11.22	42.43 ± 10.48	45.68 ± 10.38	0.254
BMI (kg/m^2^)	24.35 ± 2.45	23.44 ± 1.82	24.54 ± 2.58	25.02 ± 2.75	0.080
Gender (male)	39 (54.9%)	11 (47.8%)	11 (47.8%)	17 (68.0%)	0.264
Gender (female)	32 (45.1%)	12 (52.2%)	12 (52.2%)	8 (32.0%)	
**Baseline outcomes**					
Pain during gait	3.9 ± 1.49	3.91 ± 1.81	3.61 ± 1.20	4.16 ± 1.43	0.448
Pain during contraction	4.71 ± 1.52	4.88 ± 1.79	4.61 ± 1.20	4.88 ± 1.79	0.779
Kinesiophobia	29.86 ± 4.95	29.74 ± 5.47	28.87 ± 4.67	30.88 ± 4.68	0.374
Fatigue	11.83 ± 6.13	11.78 ± 6.47	12.78 ± 5.58	11.00 ± 6.42	0.609
ROM	15.9 ± 3.06	15.87 ± 2.65	16.39 ± 2.81	15.48 ± 3.63	0.593

Ma, myofascial.

Table 2 presents the descriptive and inferential statistics for all outcome variables, including pre- and post-intervention mean values, mean intra-group changes with their 95% confidence intervals, and between-group comparisons expressed with the corresponding effect sizes. In all variables, the intra-group analyses revealed statistically significant changes, as the 95% confidence intervals of the mean differences did not include zero. These results confirm that each intervention produced relevant modifications over time. The table also provides the effect sizes for the between-group comparisons, allowing the magnitude of differences between interventions to be examined in detail.

**Table 2:** Intra and between groups differences in outcome measures.

**Group**	**Pre**	**Post**	**Intra-group differences**	**Between-groups differences**
**Pain during gait**				
High-intensity PE	3.91 (1.807)	0.82 (1.65)	−3.09 (−3.8 to −2.37)	High vs. Low 0.22 (−0.50 to 1.00); *g* = 0.15; *p* =1.000.
Low-intensity PE	3.61 (1.20)	0.74 (1.32)	−2.87 (−3.44 to −2.3)	Low vs. Sham 0.80 (−0.20 to 1.80); *g* = 0.55; *p* = 0.173
Sham PE	4.16 (1.43)	2.08 (1.26)	−2.08 (−2.6 to −1.56)	**High vs. Sham 1.01 (0.01 to 2.00);** ***g*** **=** **0.70;** ***p*** **=** **0.049**
**Pain during contraction**				
High-intensity PE	4.61 (1.56)	0.65 (1.58)	−3.96 (−4.64 to −3.27)	High vs. Low 0.21 (−0.50 to 0.90); *g* = 0.14; *p* = 1.000
Low-intensity PE	4.61 (1.20)	0.87 (1.21)	−3.74 (−4.26 to −3.21)	Low vs. Sham 1.06 (−0.05 to 2.15); *g* = 0.67; *p* = 0.62^*^
Sham PE	4.88 (1.79)	2.20 (1.77)	−2.68 (−3.41 to −1.95)	**High vs. Sham 1.28 (2.25 to 2.30);** ***g*** **=** **0.81;** ***p*** **=** **0.017**
**Kinesiophobia**				
High-intensity PE	29.74 (5.47)	26.57 (5.47)	−3.17 (−4.25 to −2.1)	High vs. Low 0.3 (−0.8 to 1.40); *g* = 0.12; *p* = 1.000
Low-intensity PE	28.87 (4.67)	26.00 (4.67)	−2.87 (−3.79 to −1.94)	Low vs. Sham −0.25 (−1.30 to 0.80); *g* = −0.10; *p* = 1.000
Sham PE	30.88 (4.68)	27.76 (4.68)	−3.12 (−4.26 to −1.98)	High vs. Sham 0.05 (−1.00 to 1.10); *g* = 0.02; *p* = 1.000
**Fatigue**				
High-intensity PE	11.78 (6.47)	5.17 (3.33)	−6.61 (−8.05 to −5.17)	High vs. Low 0.3 (−1.87 to 2.47); *g* = 0.08; *p* = 1.000
Low-intensity PE	12.78 (5.58)	5.87 (3.94)	−6.91 (−8.62 to −5.21)	**Low vs. Sham –2.71 (–5.05 to –0.37);** ***g*** **=** **–0.78;** ***p*** **=** **0.024**
Sham PE	11.00 (6.42)	6.80 (3.00)	−4.20 (−5.44 to −2.96)	High vs. Sham −2.41 (−4.85 to 0.03); *g* = −0.69; *p* = 0.053^*^
**ROM**				
High-intensity PE	15.87 (2.65)	9.74 (2.65)	−6.13 (−6.72 to −5.54)	High vs. Low −0.83 (−2.10 to 0.44); *g* = −0.38*p* = 0,751^a^
Low-intensity PE	16.39 (2.81)	11.09 (2.81)	−5.30 (−6.37 to −4.24)	Low vs. Sham 0.34 (−0.90 to 1.58); *g* = −0.15; *p* = 1.000^a^
Sham PE	15.48 (3.63)	9.84 (3.63)	−5.64 (−6.6 to −4.68)	High vs. Sham −0.49 (−1.80 to 0.82); *g* = −0.23; *p* = 1.000^a^

Data are expressed as mean (SD) for pre- and post-intervention values and as mean (95 % CI) for mean differences. Intra-group comparisons were analyzed using paired t-tests or Wilcoxon tests, and between-group comparisons using one-way ANOVA or Kruskal–Wallis with Bonferroni/Dunn *post hoc* correction. Effect sizes (Hedges' g) and adjusted *p*-values are shown. *p* < 0.05 was considered significance.

^a^Significance has been considered based on Dunn's non-parametric test.

High, high-intensity PE. Low, low-intensity PE. Sham, sham PE.

^*^Close to significance.

Bold values indicate statistically significant between-group differences (*p* < 0.05).

### 3.2 Pain during gait and contraction

For both pain-related variables (gait and contraction), the high-intensity PE group demonstrated larger reductions compared to the sham group (*g* = 0.70; 95% CI 0.01–2.00, and *g* = 0.81; 95% CI 0.25–2.30, respectively), exceeding the predefined minimal clinically important difference (MCID) of a ≥2-point reduction on the NPRS (40). These findings suggest clinically meaningful improvements rather than mere statistical differences. Although the comparison between low-intensity PE and sham PE for pain during contraction did not reach statistical significance (*p* = 0.062; *g* = 0.67; 95% CI −0.05 to 2.15), the effect size indicated a moderate potential impact. No relevant differences were found between the two active treatment groups. These results support the likely clinical relevance of high-intensity PE in reducing pain in chronic medial gastrocnemius injuries (Table 2).

### 3.3 Kinesiophobia

No significant between-group differences were observed (Table 2). Although slight post-intervention reductions were noted across all groups, the effect sizes were negligible (g ≤ 0.12) and the 95% confidence intervals overlapped zero, indicating that these changes were not clinically meaningful.

### 3.4 Triceps surae muscle fatigue

The low-intensity PE group showed a clinically meaningful reduction in muscle fatigue compared to the sham group (*g* = −0.78; 95% CI −5.05 to −0.37; *p* = 0.024), exceeding the predefined minimal clinically important difference (MCID) of a ≥3-repetition improvement on the fatigue test, based on established thresholds in musculoskeletal pain populations (42). The high-intensity PE group also presented a moderate effect (*g* = −0.69; 95% CI −4.85 to 0.03; *p* = 0.053), suggesting a probable beneficial tendency that did not reach statistical significance. These findings indicate that both PE protocols may contribute to improving fatigue tolerance, particularly the low-intensity application, although further studies are required to confirm these results.

### 3.5 ROM

No significant between-group differences were observed in ROM. Effect sizes were small (g ≤ 0.38) and 95% confidence intervals overlapped zero, suggesting minimal clinical impact. This finding indicates that short-term improvements in ankle dorsiflexion may depend more on general rehabilitation effects than on PE dosage.

## 4 Discussion

This study evaluated the effect of PE, applied at different intensities, on relevant clinical variables in patients with subacute to early chronic medial gastrocnemius muscle injury, including pain, kinesiophobia, muscle fatigue, and range of motion (ROM) in ankle dorsiflexion. The results suggested that high- and low-intensity PE protocols may generate different patterns of improvement in the variables analyzed, especially in pain and fatigue.

It is important to interpret these findings within the context of the injury stage studied. Participants presented lesions with a duration of 3–8 weeks, corresponding to subacute and early chronic remodeling phases rather than long-standing mature fibrosis. Although fibrotic remodeling processes are known to begin within this period, histological characteristics may vary across individuals, potentially introducing biological heterogeneity. Therefore, the observed effects may be related to modulation of early fibrotic remodeling processes rather than reversal of fully established and structurally stabilized scar tissue.

Taken together, these findings indicate that PE could represent a useful intervention in the treatment of subacute to early chronic muscle injuries, although further studies are needed to confirm its clinical efficacy and determine the optimal application parameters.

In terms of pain, the application of high-intensity PE was associated with a significant reduction both during gait and muscle contraction, with moderate to large effect sizes. Improvements were also observed in the low-intensity PE group, although without reaching statistical significance. These results are consistent with previous studies reporting the analgesic potential of PE compared to sham interventions and other invasive techniques, such as dry needling (48, 49). At the physiological level, most of the proposed mechanisms are based on preclinical or animal models, where galvanic current has been shown to promote tissue regeneration and modulate inflammation by influencing the expression of genes related to repair and angiogenesis, as well as promoting the release of anti-inflammatory cytokines (25, 27, 50–52). These findings provide a biological rationale but should be interpreted with caution, as the same molecular pathways may not fully translate to human tissue. Such responses may help explain the clinical improvement in pain-related outcomes, particularly in muscle injuries undergoing subacute to early chronic remodeling processes, where fibrotic changes are actively developing and conventional techniques may sometime be insufficient to modulate tissue adaptation. Although the precise mechanisms remain incompletely understood, some authors have hypothesized the possible modulation of pain induced by EP (36). In addition, it has been reported that the application of galvanic current may cause transient vasodilation in medium- and small-caliber vessels, which might facilitate the recruitment of inflammatory cells essential for repair, as well as the clearance of accumulated nociceptive substances (18), potentially contributing to the analgesic effect observed.

Regarding kinesiophobia, no significant differences were observed between groups, although all groups showed improvement after the intervention, including the sham PE group. This pattern suggests that the reduction in kinesiophobia might be influenced by non-specific factors, such as progressive exposure to movement, the standardized intervention framework, or placebo responses. Most participants exhibited low or moderate levels of kinesiophobia at baseline, which is common in patients with chronic pain (53). Although PE may indirectly contribute to reducing kinesiophobia through functional improvement and pain relief, its direct effect does not seem to be superior to that of other therapeutic strategies. Previous studies have suggested that combining invasive techniques with pain neuroscience education programs is more effective in addressing the cognitive-behavioral component of fear of movement (54, 55). Additionally, patient expectations, verbal suggestion, and associative learning processes could have played a role in these changes (56–58).

With respect to muscle fatigue, significant between-group differences were observed in the heel-raise-test, with the low-intensity PE group showing a significant improvement compared to the sham group, and a moderate-to-large effect size. The high-intensity PE group also showed a moderate effect and a trend toward significance, suggesting a potential beneficial influence. These findings may reflect the anti-inflammatory effects attributed to PE, including possible downregulation of pro-inflammatory cytokines such as IL-6 and TNF-α and the upregulation of growth factors like VEGF and IGF-1, which support angiogenesis and muscle regeneration (24, 59, 60). Previous studies conducted in athletes with chronic soleus muscle injuries, including female soccer players and dancers, have also reported improvements in muscle fatigue following PE, although without significant between-group differences (31, 35). These discrepancies in findings may related to the specific characteristics of the muscle involved, as the medial gastrocnemius is more susceptible to fatigue induced by eccentric exercise compared to the soleus, due to its predominantly glycolytic fiber composition (61).

Regarding ROM, no significant between-group differences were observed, although all showed improvements after treatment. This improvements may be due to the standardized pre-treatment procedures or to mechanical effects of the needling itself, which has been shown to induce adaptive responses at the level of the extracellular matrix and the central nervous system (CNS) (48, 62, 63). At the CNS level, the mechanical stimulation generated by the needle might influence the activity of sensory receptors and modulate spinal circuits, potentially contributing to adaptive changes in muscle tone regulation. While studies such as Albin et al. (64) have reported improvements in stiffness and ROM following dry needling, others such as Benito-de-Pedro et al. (65) have found no significant differences between dry needling and ischemic pressure techniques. As for PE particularly, previous work has shown benefits in ROM in myofascial conditions, although these effects are not consistently observed in subacute to early chronic muscle injuries (35, 65, 66). This suggests that the dosages and parameters applied may not have been sufficient to induce substantial structural reorganization during extracellular matrix remodeling, potentially explaining the lack of between-group differences. Nevertheless, given the absence of imaging-based follow-up and the short-term nature of the assessment, it cannot be definitively established whether structural remodeling took place.

### 4.1 Limitations

This study has several limitations that should be considered when interpreting the results. First, the follow-up period was limited to 28 days, restricting the assessment to short-term effects of PE. No data on medium- or long-term outcomes were collected; therefore, future research should extend the follow-up period to evaluate the persistence of benefits or the occurrence of potential relapses. Additionally, a final assessment was conducted only after completion of the intervention protocol. Although this design aligned with the aim of the study, including intermediate assessments would have been useful to examine the trajectory of clinical changes. This issue could be addressed in future studies adopting a more longitudinal approach.

Second, all participants underwent the same standardized pre-treatment before the intervention sessions. While this approach ensured consistency across groups, it may also have influenced the results, potentially reducing the specificity of the observed effects attributable to PE.

Another limitation was the difficulty in recruiting patients with sufficiently advanced lesions to confirm fibrosis, which reduced the sample size. Moreover, although all participants presented injuries with a duration of 3–8 weeks, this time range may involve relevant histological differences between subjects, potentially affecting the homogeneity of the groups.

Regarding blinding, participants were informed that they might or might not perceive current during treatment. No evidence suggested that blinding was compromised; however, the effectiveness of patient blinding was not formally assessed. Additionally, due to the inherent characteristics of the intervention, the treating clinician could not be blinded, and therefore the effective study design corresponds to a single-blind randomized controlled trial. Although outcome assessors and participants were blinded, the absence of therapist blinding may introduce a potential risk of performance bias. Therefore, some influence of the procedure during treatment administration cannot be entirely ruled out.

Another limitation of the present study is the absence of more functional outcome measures and imaging-based follow-up assessments. The inclusion of objective imaging techniques, such as elastography, quantitative ultrasound analysis, or magnetic resonance imaging, could have provided more precise characterization of the degree of fibrotic remodeling and reduced potential biological heterogeneity across participants. Furthermore, imaging-based follow-up would have allowed evaluation of structural changes in the affected tissue and their relationship with the observed clinical improvements.

Finally, the limited existing literature on the use of PE for subacute to early chronic medial gastrocnemius muscle injuries makes direct comparison with other studies challenging, especially given that most previous research has focused on acute conditions or different anatomical regions. Despite these limitations, the study's design, implementation, and the consistency of the results with similar research support the validity of the findings presented.

### 4.2 Future perspectives

To date, no studies have specifically evaluated the effect of PE on muscle scar tissue, underscoring the need for and relevance of the present study. The results obtained represent a step forward in understanding the applicability of this technique, providing preliminary evidence of its potential to reduce pain and muscle fatigue in patients with subacute to early chronic medial gastrocnemius injuries.

From a clinical perspective, these findings suggest that PE could be considered as an adjunct option within multimodal rehabilitation programs for chronic muscular fibrosis, particularly in patients who show limited progress with conventional treatments. However, clinical implementation should remain cautious until further confirmatory evidence is available.

One of the most promising directions is the exploration of the dose-dependent effect of galvanic current, since higher intensities could induce a deeper reorganization of scar tissue. Future research should aim to optimize the parameters of galvanic current application and clarify possible dose-related effects, as higher intensities might induce more pronounced remodeling of fibrotic tissue. Moreover, future studies should include more specific inclusion criteria regarding lesion type and duration to better control for chronicity-related variability and enhance the accuracy of treatment effect interpretation. Importantly, medium- and long-term follow-up assessments (at least 3–12 months) are necessary to determine the durability of clinical improvements and to evaluate potential recurrence rates.

It would also be relevant to evaluate PE in patients with longer-lasting injuries (>2 months), where advanced fibrosis remains a therapeutic challenge.

Overall, this study represents an initial step toward understanding the role of PE in the management of muscle injuries involving fibrosis and provides a foundation for future research focused on its long-term efficacy, structural effects, and clinical applicability.

## 5 Conclusions

High-intensity percutaneous electrolysis (PE) was more effective than the sham intervention in reducing pain during gait and muscle contraction, while the low-intensity modality showed improvements that did not reach statistical significance. No differences in kinesiophobia and range of motion were observed between the groups. A significant decrease in muscle fatigue was observed in the low-intensity PE group compared to sham group. Although no significant interaction between intensity and outcomes was found, the differential response patterns observed across groups should be interpreted as exploratory findings rather than evidence of a confirmed dose-dependent effect. Further studies are required to verify these trends and clarify potential intensity-related responses.

## Data Availability

The raw data supporting the conclusions of this article will be made available by the authors, without undue reservation.
